# Germinal Matrix-Intraventricular Hemorrhage of the Preterm Newborn and Preclinical Models: Inflammatory Considerations

**DOI:** 10.3390/ijms21218343

**Published:** 2020-11-06

**Authors:** Isabel Atienza-Navarro, Pilar Alves-Martinez, Simon Lubian-Lopez, Monica Garcia-Alloza

**Affiliations:** 1 Division of Physiology, School of Medicine, Universidad de Cadiz, 11003 Cadiz, Spain; isabel.atienza@uca.es (I.A.-N.); pilar.alvesmartinez@alum.uca (P.A.-M.); 2 Instituto de Investigacion e Innovacion en Ciencias Biomedicas de la Provincia de Cadiz (INiBICA), 11009 Cadiz, Spain; 3 Division of Paediatrics, Section of Neonatology, Hospital Universitario Puerta del Mar, 11009 Cadiz, Spain

**Keywords:** germinal matrix-intraventricular hemorrhage, preterm newborn, neuroinflammation, microglia

## Abstract

The germinal matrix-intraventricular hemorrhage (GM-IVH) is one of the most important complications of the preterm newborn. Since these children are born at a critical time in brain development, they can develop short and long term neurological, sensory, cognitive and motor disabilities depending on the severity of the GM-IVH. In addition, hemorrhage triggers a microglia-mediated inflammatory response that damages the tissue adjacent to the injury. Nevertheless, a neuroprotective and neuroreparative role of the microglia has also been described, suggesting that neonatal microglia may have unique functions. While the implication of the inflammatory process in GM-IVH is well established, the difficulty to access a very delicate population has lead to the development of animal models that resemble the pathological features of GM-IVH. Genetically modified models and lesions induced by local administration of glycerol, collagenase or blood have been used to study associated inflammatory mechanisms as well as therapeutic targets. In the present study we review the GM-IVH complications, with special interest in inflammatory response and the role of microglia, both in patients and animal models, and we analyze specific proteins and cytokines that are currently under study as feasible predictors of GM-IVH evolution and prognosis.

## 1. The preterm newborn

A preterm newborn (PTNB) is the one born before 37 weeks gestation, considering that a normal gestation lasts for 280 ± 15 days [1]. Depending on the gestational age, PTNB can be classified as extremely preterm (<28 gestational weeks), very preterm (28 ≥ 32 gestational weeks), moderately preterm (32–33 gestational weeks) and late preterm (34–36 gestational weeks) [2,3]. In addition, according to the weight at birth, newborns can be classified into low weight (<2500 g), very low weight (<1500 g) and extremely low weight (<1000 g) [4]. At present, nearly all neonatal deaths occur in PTNB [1,5] and mostly depend on the gestational age and the birth weight [1,5]. Therefore, shorter gestational age and lower body weight are associated with an increased risk of developmental delay [3,6]. Although body weight is often used as an indicator of gestational age, both concepts should not be freely interchangeable. Whereas gestational age is a preferred criterion [6], the difficulty to correctly establish the gestational age in many cases, often makes body weight the most widely used approach [7].

There are 15 million premature births worldwide every year, accounting for ≈11.1% of all births [2,8], that are responsible for ≈3.1 million neonatal deaths per year. Therefore, preterm birth is the leading cause of death in children, accounting for 18% of all deaths among kids under 5 years old, and as much as 35% of all deaths among newborns (aged <28 days) [9]. Moreover, the lack of information from developing countries, where antenatal and perinatal cares are limited [4], is probably hampering a more accurate estimation [2]. Globally, the incidence of premature births has increased by 1% in the last 10 years [1,2]. However, the incidence of preterm births varies significantly depending on the geographic region, being higher in lower-income countries (11.8%) [2], followed by low-middle income countries (11.3%) and middle and high income countries (9.3% and 9.4%, respectively) [2]. In most developed countries PTNB represent 5–7% of births, except in the USA where premature births account for 10–12% of total births [4,10], whereas the highest rate of premature births (over 60% of all births) are detected in sub-Saharan Africa and South Asia countries [2].

### 1.1. Etiology and Consequences of Prematurity

Premature deliveries might be spontaneous deliveries (unexplained preterm labor or spontaneous rupture of the amniotic membranes) or deliveries caused by medical reasons [2,11]. Nevertheless, it has been reported that up to 20% of induced preterm births are based on clinical experience without a specifically justified medical indication, and whereas some studies have reported that purely elective preterm births might be under 10%, other studies have reported over 50% of non spontaneous late preterm births were non-evidence based [12,13,14]. Moreover, one in five cases could have reached a higher gestational age with fewer future complications [12]. In France and the USA, nearly 42% of all cesarean sections are performed when the fetus is growing poorly, increasing the birth rate and survival of the PTNB. In contrast, in developing countries lacking the means and tools, pregnancies will follow their natural course with much lower percentages of induced deliveries [2]. The majority of spontaneous premature births are due to intrauterine infections followed by maternal smoking, unfavorable economic situations and multiple gestation [4]. The increased use of assisted reproduction techniques in recent years also results in multiple pregnancies, 50% of which will be premature [1,11]. Premature birth is associated with complications that can extend from childhood to adulthood, resulting in high economic and societal burdens [11,15,16]. In addition, PTNB have an greater risk of developing associated pathologies, mainly respiratory (respiratory distress syndrome and bronchopulmonary dysplasia) [16,17], cardiological (patent ductus arteriosus) [18] and neurodevelopmental [3] disorders. Most relevant morbidities, associated with a higher risk of mortality in extremely PTNB include severe germinal matrix-intraventricular hemorrhage (GM-IVH), respiratory distress syndrome and necrotizing enterocolitis [19]. Also, a recent study on PTNB with a gestational age of 28.8 ± 2.9 weeks reports that despite the advances in modern neonatology the incidence of severe IVH, necrotizing enterocolitis and periventricular leukomalacia remained stable between 2001 and 20116 [20]. Interestingly, perinatal and, most relevantly, environmental factors, including from maternal education and occupation, whether they kids are taken care by the parents, stress exposure during neonatal intensive care unit stay or malnutrition among others, might have the greatest influence on future neurodevelopmental delay [3,16].

### 1.2. Neurological Complications of the PTNB

Even though the mortality of the PTNB has decreased, the fact that extremely PTNB have better survival rates also means that the incidence of neurological complications has increased, including neurodevelopmental and functional disorders [6,21]. Concretely, extremely premature and extremely low birth weight infants are born at a critical time in brain maturation, and improving neurological development outcomes remains a challenge. In normally growing fetuses, between 25 to 37 weeks of gestation total brain volume increases by 230%, brain stem volume increases by 134% and the cerebellum volume increases by 384% [15]. Other authors describe that the volume of the cerebellum increases fivefold between weeks 24–40 [22]. Besides, from 24 weeks gestation on, cortical gray matter matures, radial glia disappear, the complexity of the connections increases, and cortical folding and gyrification become progressively more complex. In the white matter there is a major development of axons, glial cells and oligodendrocytes [15]. Altogether, these data stress the relevance of impaired brain development, size, structure, connectivity and function in the PTNB at this stage [15]. Central nervous system (CNS) immaturity is based, among others, on the fragile vascular structure of germinal matrix (GM), low neuronal migration, poor myelinization of white matter and exponential growth of the grey matter [1]. These limitations result in different types of brain lesions in the PTNB, including white matter injury (usually associated with neuronal and axonal disturbances in cerebral cortex and other gray matter areas), intracranial hemorrhages (including GM, intraventricular and intraparenchymal), cerebellar injury [5,15,23,24], periventricular leukomalacia, periventricular hemorrhagic infarction with subsequent posthemorrhagic hydrocephalus [25] or posthemorrhagic ventriculomegaly [21].

## 2. Germinal Matrix-Intraventricular Hemorrhage

GM-IVH represents the most important neurological complication of the PTNB [26,27]. It is the most common intracranial hemorrhage, whereas subdural and subarachnoid hemorrhages are less frequent [7]. Technological advances in neonatal intensive care and perinatal medicine have significantly increased survival rates of PTNB suffering GM-IVH [28,29], especially in extremely PTNB [30]. Nevertheless, increased survival rates are accompanied by an increase of GM-IVH morbidity [31] since GM-IVH is responsible of severe complications in the majority of PTNB [32]. GM-IVH is caused by the rupture of GM vessels. The GM is a highly vascularized structure located in the periventricular subependymal region and a source of neuronal and glial cells in the immature brain, that will migrate during fetal brain development [5,32]. These glial precursors will constitute in oligodendrocytes, white matter astrocytes and GABAergic neurons in the thalamus and cerebral cortex [5]. Initially the GM surrounds the whole fetal ventricular system, and begins to regress at 28 weeks until it disappears at full term [29,32]. It has been described that the GM starts to involute after 32 gestational weeks and consequently, the risk of hemorrhage decreases from that time on [7]. However, in the premature brain (<32 weeks of gestation) the cerebral white matter is occupied by premature oligodendrocytes and oligodendrocyte precursor cells, which are much more sensitive to excitotoxicity and oxidative stress than mature oligodendrocytes [33]. In very low birth weight babies, approximately at the time of birth, glial precursors are migrating into the cerebral cortex [23] and alterations in this moment may result in a deficit of oligodendroglia and astrocytic precursor cells that can affect myelinization and cortical development [15,23,34].

GM-IVH can also exceptionally occur in full term newborns and may be due to maternal risk factors [23,35,36] or severe asphyxiation during childbirth [37], accounting for 3–5% of cases (Matijevic et al., 2019). However, in full-term newborn, most GM-IVH originates in the choroid plexus and less frequently in the GM, unlike the PTNB [38]. Therefore, GM-IVH occurs almost entirely in PTNB, especially those born with <1500 g and/or <32 weeks gestation, who are very vulnerable to ischemia and bleeding [7] due to their immature CNS, hemodynamic instability [38], difficulty to autoregulate cerebral blood flow and compensate the fluctuations [1] or extreme sensitivity to hypoxia and changes in osmolality and tension [1]. As a consequence, when there are changes in blood pressure, arterial carbon dioxide partial pressure and cerebral blood flow, PTNB cannot compensate for these variations and fragile GM vessels can easily break [39], causing bleeding from the GM [34]. The fragility of the GM in the PTNB is due, among others, to: (i) a capillary network with large vessels and weak endothelial walls [5], (ii) vessels with an endothelial layer with few pericytes [24] because of reduced signaling of tumor growth factor 1 (TGF-1), (iii) an unstable basal lamina as a consequence of a decrease of fibronectin expression [27] and collagen deficiency, (iv) a blood-brain barrier (BBB) with discontinuous astrocyte prolongations [5], (v) a weak structure of the cytoskeleton that supports blood vessels caused by a limited glial fibrillar acid protein expression in the astrocytes end-feet, affecting the mechanic resistance of blood vessels [5,24], or (vi) a vasculature lacking self-regulation mechanisms to modulate blood vessels light under fluctuating hemodynamic conditions [5]. Furthermore, GM has a rich terminal vascularization with an intense metabolism [34] that predisposes to vessel rupture of the subependymal area [24,34].

Normally, the GM-IVH originates in the first days of life [15,40], and rarely occurs during birth [41]. In more than 90% of cases GM-IVH appears in the first week after birth [40], being rare the cases in which the GM-IVH occurs after the third day of life [21]. GM-IVH may spread the next days, block the venous drainage of the terminal veins affecting the adjacent parenchyma and developing ventriculomegaly by obstruction of cerebrospinal fluid (CSF) circulation [32]. Typically, GM-IVH is categorized into 4 degrees according to the severity of the GM-IVH [42,43]. Grades I and II lesions are known as mild GM-IVH, and grades III and IV lesions are considered severe GM-IVH [44].

- Grade I: hemorrhage localized only in the subependymal GM (caudo thalamic groove).
- Grade II: intraventricular hemorrhage without ventricular dilation.
- Grade III: intraventricular hemorrhage with ventricular dilation.
- Grade IV: parenchymal hemorrhage that corresponds to periventricular venous infarctions with hemorrhagic evolution [5].

**Figure 1:**
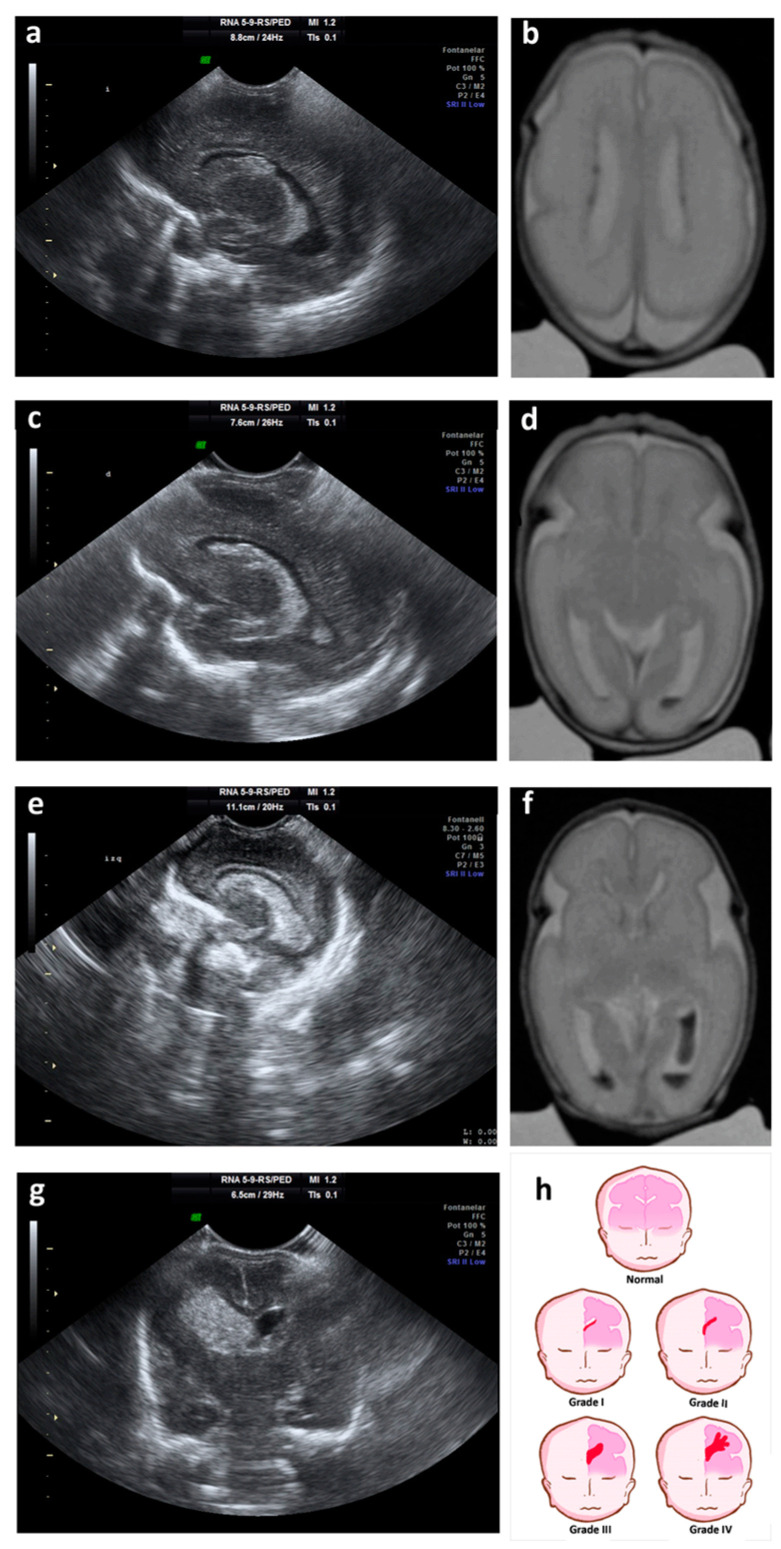
**Ultrasound and magnetic resonance imaging (MRI) showing different grades of GM-IVH.** (**a**) Parasagittal cerebral ultrasound through lateral ventricles shows grade I hemorrhage; (**b**) T2-weighted axial MRI shows grade I hemorrhage on both lateral ventricles; (**c**) parasagittal cerebral ultrasound through lateral ventricles shows grade II hemorrhage; (**d**) T2-weighted axial MRI shows grade II hemorrhage on the left lateral ventricle; (**e**) parasagittal cerebral ultrasound through lateral ventricles shows grade III hemorrhage; (**f**) T2-weighted axial MRI shows grade III hemorrhage on the left lateral ventricle and grade II hemorrhage on the right lateral ventricles; (**g**) coronal cerebral ultrasound shows grade IV or periventricular hemorrhagic infarction; (**h**) cartoon representing ultrasound classification of the germinal matrix-intraventricular hemorrhage (GM-IVH).

Currently, grade IV is no longer considered a propagation of the original hemorrhage, but a consequence of the obstruction of the venous drainage, with a consequent venous infarction and an hemorrhage of the adjacent tissue (peri-ventricular hemorrhage infarction) [25] (Figure 1). This, among other reasons, has caused other classifications to be proposed for grading the severity of the GM-IVH [45].

In many cases GM-IVH and peri-ventricular hemorrhage infarction are clinically silent and detected during routine cranial ultrasound. Some infants present subtle changes in the level of consciousness, limb movement, tone, and eye movement in the hours to days after the GM-IVH. With extensive hemorrhage, a catastrophic deterioration occurs with stupor, “decerebrate” posturing, generalized tonic seizures and hypotonia [41]. 

### 2.1. GM-IVH Neurodevelopmental Disabilities

As previously stated, improvements in medicine and neonatal care have increased survival rates of the PTNB [46] that also translates to a higher burden of associated disabilities [1,5,23]. PTNB exchange the protective environment of the womb for the stress of the neonatal intensive care unit, with a multitude of physical and sensory stimulations for which they are unprepared. In addition, when newborns are separated from their mothers, they do not receive the necessary biological and maternal emotional care, which triggers adverse short and long-term developmental consequences, including structural and functional alterations of brain development and dysregulation of the hypothalamic-pituitary-adrenal axis stress response system [47] among others. Moreover, brain abnormalities are directly related to an increased risk of sensory [7,23,35], cognitive and motor [33,41] impairments. PTNB with severe GM-IVH commonly suffer developmental delay [23,35,48], associated to cerebelum abnormalities [22], as well as a consequent cerebral palsy [7,23]. Previous studies have reported that approximately 10% of PTNB with severe GM-IVH will develop cerebral palsy [49] and these children will frequently suffer spastic dysplasia where both legs are affected [5]. Nevertheless, other studies have also shown that very PTNB with periventricular hemorrhage develop cerebral palsy in up to 42% of the cases [50], showing that different studies report inconsistent outcomes, and supporting the difficulty of these assessments. Other alterations include visual impairment and hearing loss, that may affect up to 3% of the todlers. Also, recent studies have reported that 15.6% of children had visual deficieny and 7.8% presented hearing impairment [34]. Other studies have reported visual problems associated to the severity of the IVH (ranging from 26.1% in grade I IVH up to 45.5% in grade III IVH) without much effect on acoustic impairment [51] and a recent review and methaaanysis has reported no visual or hearing impairment after periventricular hemorrhage [10]. In addition, extremely premature and very underweight children have deficits in intellectual quotient, expressive and receptive language skills or spatial reasoning [15,16]. Thus, it has been described that GM-IVH increases by twofold the need of special education in very preterm and very low body weight infants with mild GM-IVH, compared to PTNB without GM-IVH [23]. Also, Mukerji et al. have described deficits in academic performance for PTNB with mild and severe GM-IVH [29]. Likewise, a study by Holwerda et al. indicated that school-aged PTNB with grade III GM-IVH had intelligence, visual perception, attention and emotional functioning alterations when compared to PTNB without GM-IVH. Nevertheless, these authors did not detect any disabilities when visual-motor integration, verbal memory and executive functioning were evaluated [48]. In addition, several studies describe that PTNB (including late PTNB) are at higher risk than full-term newborns of suffering neuropsychiatric problems such as autism spectrum disorders, attention deficit hyperactivity disorders, anxiety, depression and antisocial behavior [3,52]. Interestingly, PTNB with periventricular venous hemorrhagic infarction showed worse motor alterations than cognitive problems [48]. Although severe GM-IVH is undoubtedly associated with impaired neurodevelopment [23,29], the outcome of low-grade GM-IVH has not yet been agreed upon [10,33] and it has been suggested that neurodevelopmental alterations in these patients might be exclusively associated to prematurity [29]. Nevertheless, Radic et al. describe that disabilities associated to GM-IVH are highly dependent on the severity of the lesions [26], and in line with these observations it has also been reported that even lower degrees of GM-IVH of the PTNB predisposes to neurological complications, such as cerebral palsy and developmental delay [26,53]. Previous studies have shown that there are no significant differences in the short term when neurological development and cerebral palsy are compared between low-grade GM-IVH and non-GM-IVH controls [36]. Nevertheless, follow-up must continue until school age as significant differences have been detected up to age 16 [23,36] and other authors describe long-term consequences on the neurodevelopmental outcome of PTNB with low-grade GM-IVH [30].

### 2.2. GM-IVH Associated Brain Damage

After the rupture of the GM vessels, blood is deposited in the intraventricular space and red blood cells are lysed, releasing hemoglobin into the intraventricular CSF [54] and the periventricular white matter [30]. This hemoglobin is highly reactive and spontaneously self-oxidizes from oxy-hemoglobin to met-hemoglobin and superoxide ion [54]. After several reactions, heme is converted into hemosiderin, which can escape from the CSF and deposit on the brain stem and the surface of the cerebellum, damaging these structures [33] and altering the normal development of the cerebellar cortex [30]. In addition, degradation of the heme group produces bilirubin, carbon monoxide and free iron. Free iron can generate reactive oxygen species that damage lipids, proteins and DNA. It can also be inserted between cell membranes with cytolytic effects, leading to periventricular cell death [33,54]. Altogether, GM-IVH causes the loss of cell progenitors and greater white matter injury due to oxidative stress and pressure, contributing to the pathogenesis of periventricular leukomalacia [5]. Furthermore, even small hemorrhages of the GM can have negative effects on the migration of neuronal and glial cells in the brain of a premature infant [23], and higher ventricular volumes have been related to lower cognitive efficiency in the PTNB with GM-IVH [25]. GM-IVH may also be complicated by hydrocephalus [5]. When GM bleeding invades the ventricular system, CSF circulation can be obstructed and an ependymal inflammatory response may also occur, causing a decrease in CSF reabsorption and resulting in a consequent posthemorrhagic hydrocephalus [34]. 

### 2.3. Neuroinflammation and Microglia in the GM-IVH

During fetal development, neural and glial precursor cells are found at the head of the caudate nucleus, below the lateral ventricles [55]. Among the glial cells, microglia are the macrophages of the CNS [56,57], which are the first to respond to ischemia [55]. They play essential roles such as communication between cells, phagocytosis of microbes, cellular debris, apoptotic and cancerous cells, and other foreign substances. Moreover, unlike monocytes, microglia have definite effects on embryonic vasculogenesis and vascular anastomosis [58]. Interestingly, there are differences between the adult and newborn microglia [55,59]. In the developing brain, microglia has an amoebic morphology, while in the mature brain microglia has a ramified form [57]. Besides, in the adult brain they respond rapidly to injury by producing inflammatory cytokines that aggravate brain injury [58], and in the postnatal brain they prune out synapses and form synaptic circuits [59]. Likewise, during brain development, activated microglia are involved in the elimination of transcallosal projections, vascularization and angiogenesis, myelinization, programmed cell death, and axonal guidance of white matter, among others [57]. PTNB have a higher number of microglia in the periventricular white matter relative to other parts of the brain, being even higher in those areas close to the GM. Therefore, any degree of hemorrhage can activate the microglia, triggering cellular apoptosis, although the degree of activation depends on the severity of the GM-IVH [57]. Accordingly, in neonatal brains the depletion of the microglia due to the lesion results in the elimination of endogenous protective mechanisms that improve the outcome of the injury [59]. In compliance with these studies, the dual role of pro-inflammatory activated microglia after ischemic brain injury and the neuroprotective-neuroreparative role of microglia has been further taken into consideration [56,58]. While controversial, microglia state of activity may change between the classic activation state (M1 phenotype) and the alternative activation state (M2 phenotype). The M1/M2 polarization of the microglia consists of the expression of the M1 (CD68, CD86 and inducible nitric oxide synthase) and M2 (CD206, Ym1 and Arginase-1) genes [60]. The M1 phenotype is induced by lipopolysaccharides, interferon-γ, TNF-α, stimulation of Nod-like receptors or Toll-like receptors (TLR), and secretes the pro-inflammatory cytokines TNF-α, IL-1β, IL-6, IL-12, and IL-23 [56], which prevent CNS repair and spread tissue damage [60,61]. In contrast, the M2 phenotype is induced by IL-4, IL-10, TGF-β, IL-13, and secretes anti-inflammatory cytokines IL-10 and TGF-β [55,56], promoting an anti-inflammatory response [56] that resolves local inflammation and eliminates cellular debris, eventually recovering the brain [60,61]. Therefore, the decrease in microglia and, consequently, the deficit of anti-inflammatory cytokines together with the increase in the astrocytic reaction are associated with a more severe injury [55]. For this reason, it would be interesting to investigate the attenuation of the M1 phenotype to favor the activation of the M2 phenotype, as suggested in preclinical studies [56,59]. Despite of this, the hypothesis of M1/M2 phenotypes has limitations, as more and more subsets of microglia phenotypes are found. This may mean that M1/M2 are the extremes of a wide range of macrophage/microglia subsets in which each one plays a critical immunomodulatory role in the GM-IVH [56]. 

After GM-IVH, the hematoma applies mechanical pressure to the glia and neurons [59], cytotoxic edema and necrosis, known as primary lesion [56]. The secondary lesion results of the entry of blood components into the brain tissue and the resident and peripheral immune cells that trigger the secretion of pro-inflammatory mediators, extracellular proteases and reactive oxygen species, together with an alteration of BBB [56]. Previous studies have shown that blood-derived products, such as thrombin and plasminogen, can contribute to the activation of microglia with the consequent release of inflammatory cytokines that damage the adjacent white matter [57]. Extracellular hemoglobin metabolites also have pro-inflammatory effects on endothelial cells and macrophages, and may even activate innate immunity, acting as ligands of the TLR system [54]. As a consequence, inflammatory fibrosis or arachnoiditis with gliosis may be triggered, promoting an imbalance in CSF production, absorption or transit [62]. In addition, the microglia is responsible for antigen presentation [55] as well as phagocytosis at the site of the hematoma and in the adjacent damaged or dead tissue [59,61]. Microglia also promotes astrogliosis [63], which contributes to cytotoxicity and necrosis [59], while inflammation mediators such as interleukins IL-1β, IL-6, TNF and matrix metalloproteinases [55] further damage the tissue [56]. 

Whereas previous studies have not been able to confirm a link between inflammation and hemorrhage [64], other studies have found pro-inflammatory cytokines in the fetus that can predict the risk of GM-IVH [64,65,66]. Pro-inflammatory fetal cytokines (TNF, IL-6) have been detected and a direct relationship has been found between umbilical cord IL-6 concentrations and GM-IVH [66]. Likewise IL-1 and IL-8 are increased in children with cerebral palsy, along with vascular endothelial growth factor (VEGF) [65]. However, CCL-18 chemokine levels are low in the umbilical cord of newborns, while CCL-18 levels increase from 32 weeks of gestation, as the risk of hemorrhage decreases [64]. CCL-18 receptors are found in the choroid plexus, periventricular capillary endothelium, ependymal cells and the GM. Therefore, it has been suggested that CCL-18 low levels can predict the risk of a grade II-IV GM-IVH [64]. Also XCL-1 has been reported to be reduced in the CSF from hydrocephalic PTNB, while CCL-19 is significantly increased [62].

## 3. Animal Models of GM-IVH

The severity and complications associated to GM-IVH [67] have opened the door to the development of animal models to further understand the neuropathological features as well as to explore new therapeutic options [68]. Rabbits [63], dogs [69], lambs [70], sheep [71], rats [72], mice [32] or pigs [73] have been previously used to study GM-IVH of the PTNB. However, rodents are the most commonly used models. Mice and rats are regularly used because their brain development is well known [74] and their neuroanatomy [75,76], proliferation and differentiation processes [77,78,79,80] or synaptogenesis [81,82,83] have been studied in depth, making it possible to establish brain development comparisons with the human brain. Moreover, alterations of the GM [79,80,84], as well as motor and behavioural consequences [85,86,87], have also been studied in rodents, supporting their suitability as models to reproduce the GM-IVH of the PTNB [88,89]. Nevertheless, relevant differences need to be taken into consideration when using rodents to reproduce GM-IVH pathology; on the one hand, rodents brains at birth are more immature than the human PTNB brain. Brain development in rats at 6 days of age (P6) is equivalent to 35 weeks of gestational age in humans [90], while mice brain development at birth (P0) is equivalent to 22–24 gestational weeks in humans [84]. Besides, GM-IVH does not occur spontaneously in rodents, so transgenic mouse models have been created to induce spontaneous bleeding. In addition, intraventricular administration of glycerol to rabbits, as well as autologous blood or collagenase to rodents, is regularly used to induce GM-IVH in animals.

### 3.1. Genetically Modified Models

Whereas genetically modified animal models that reproduce a GM-IVH are limited, they provide a relevant tool to study spontaneous bleeding in the brain. To our knowledge only a few transgenic models have been developed to the study of GM-IVH, including a transgenic mouse with mutations for integrin [91], a transgenic mouse with mutations for procollagen IV [92] and mice overexpressing VEGF [93]. Interestingly, transgenic models developed to reproduce other diseases, such as cleft palate, can also present GM-IVH [94].

Integrins are heterodimeric receptors, of non-covalently associated α and β subunits, strongly expressed in the CNS [95]. Integrins link the extracellular matrix to the cytoskeleton, some soluble factors and cell surface proteins [96]. They are necessary for the postnatal migration of glial cells [97] and as adhesion molecules that mediate multicellular interactions in the BBB. Complete ablation of the gene for the αv integrin subunit causes 100% lethality of the offspring. Among these, 70% die between E9.5 and E10.5, and those that survive to E12.5 develop brain hemorrhages within the ganglionic eminence of the telencephalon. Also, null αv neonates are severely hydrocephalic and die within a few hours after birth [91,98]. Further assessment of the defects that contribute to brain hemorrhage in αv-null embryos revealed normal assembly of perycites and endothelial cells in the brain, whereas a compromise was observed when the interaction between brain microvessels and parenchyma was addressed [91]. Other studies have shown that global detection of the gene for β1 integrin specifically, also results in early embryonic death at E5 [99]. Conditional deletion of β1 integrin leads to abnormal vascular patterning and embryonic death at E9.5-E10, and whereas heterozygous endothelial β1 integrin deletion does not affect postnatal survival, it reduces β1 expression by 40% and interferes with normal vascular remodeling [99]. Deletion of αvβ3, αvβ5 or αvβ6 genes results in viable and fertile mice that do not show brain hemorrhages [91]. On the other hand, ablation of β8 integrin results in lethality rates that reach 65% by midterm, and the remaining 35% die shortly after birth. Embryos present vascular abnormalities, leaky capillaries, irregular capillary patterning and endothelial cell hyperplasia [100]. Supporting these observations, other studies have shown that induced loss of β8 expression in the GM neural progenitors, also leads to defective vessel development, region-specific vascular defects and hemorrhages similar to those observed in human GM hemorrhage [101]. Altogether, these studies show that integrins establish and maintain vascular integrity by the interaction of the micro-vessels with the cells of the brain parenchyma.

In 2005, Gould et al. developed a mouse model to study porencephaly that harbours a semidominant mutation in the procollagen type IV gene, so the secretion of type IV collagen is inhibited [92]. Interestingly, this mouse also presents spontaneous bleeding, making it appropriate as a GM-IVH model. Procollagen IV is part of the basal membrane of different tissues, including the epithelium of blood vessels and mutations in procollagen IV gene are associated with vascular problems in adults and fetuses with cerebral hemorrhages. About 50% of the heterozygous transgenic procollagen IV mice die the day of birth and approximately 18% of the survivors had porencephaly. These mutant mice also have small size, reduced viability and could have multiple pleiotropic phenotypes, as ocular anomalies, mild renal anomalies or reduced fertility [102].

Yang et al. have also developed a tetracycline-regulated transgenic (VEGF-Tet) system to check the effects of inducing VEGF in the GM. VEGF is largely implicated in angiogenesis [103] and vascular maturation. Previous clinical studies have shown a significant increase of serum VEGF levels in GM-IVH patients, when compared with control babies. Interestingly, the occurrence of GM-IVH increases as serum VEGF levels raise, and authors also reveal that CSF levels of VEGF can predict the need for permanent shunt placement [104]. In line with these observations, VEGF-Tet-Off mice initially develop a dense network of loosely adjoined endothelial cells and pericytes near the lateral ventricles that evolves to a low-vascularity periventricular area [93], reproducing the weak and immature vascular system of the PTNB [93,105] and GMH-IVH-like anomalies. As described in patients, the severity of the lesions in VEGF-Tet-Off mice ranges from bleeding with ventriculomegaly to bleeding with destruction of white matter [93]. Yang et al. also suggest that VEGF activates the induction of neurovascular proteases, including matrix metalloproteinase 9, cathepsins, and caspase-3 as feasible contributors to the lesion [93]. Whereas it is an extremely useful model to assess the causes of intracranial hemorrhage, the fact that over 80% of VEGF-Tet-Off embryos die before birth, limits the utility of this animal to assess the evolution of the lesions or to study therapeutic approaches. 

While not specifically developed to reproduce GM-IVH of the PTNB the crossing of heterozygous male mice for the Tgfb3-Cre and Alk5 knockout alleles with female mice homozygous for the floxed Alk5 allele (Alk5/Tgfb3-Cre mice) [94] results in brain complications that resemble the GM-IVH of the PTNB. Postmortem studies revealed that these mice can suffer hydrocephalus with pronounced dilation of the ventricles, as well as compression of the cerebellum due to increased volume of CSF. The results of this research show that TGF-β signaling is implicated in the maintenance of vasculature integrity within the GM [94]. 

Altogether, transgenic mouse models are currently providing new venues to study the etiology of the GM-IVH, as well as preventive possibilities. Nevertheless, they are only partially characterized, and lethality rates and/or short lifespan limit studies in the long term or neurobehavioral assessment, that are needed to fully understand GM-IVH complex pathology.

### 3.2. Lesion-Induced Models

Given the fact that spontaneous bleeding hardly occurs in animal models, GM-IVH might be induced by different approaches. Despite the pathophysiology of the lesions is largely different to that observed in PTNB, these models have the advantage of allowing the identification of the exact time and location of the bleeding. Most models use glycerol [106,107,108], blood [72,89,109,110,111] or collagenase [60,72,112] to induce GM-IVH. Whereas not strictly a drug-induced lesion, intracortical injection of phosphate-buffered saline to P3 or P5 plasminogen activator inhibitor 1 KO mice results in lesions that resemble GM-IVH, including white matter and cortical lesions as well as ventricle enlargement, accompanied by motor activity alterations. The deleterious effects are not observed when lesions are induced at P10, suggesting that microvascular maturity directly determines the outcomes in this animal model [113].

#### 3.2.1. Glycerol-Induced GM-IVH in Rabbits 

Rabbits have been largely used to reproduce GM-IVH. The model relies in the similarities between rabbits and human vascular structure of basal ganglia [106] as well as the comparable brain development. Among others, the maximal growth of the brain occurs prenatally in rabbits and humans, the structure and function of the GM are similar and the GM involutes at birth both in rabbits and humans. Also, preterm rabbits have high survival rates since the maturation of the lungs is completed just before term [107]. Studies in rabbits have shown that GM vessels in premature E28 animals present structural characteristics of a BBB. However, many ultrastructural abnormalities are observed in the vasculature: the basal lamina is thin, discontinuous and poorly defined, smooth muscle cells and collagen are absent and astrocytes are immature, supporting the incapacity of the ganglionic eminence vasculature to endure the transmural pressures and other factors that contribute to BBB instability and ultimately lead to the development of GM-IVH [114] (see Table 1). GM-IVH can develop spontaneously in rabbits, allowing the study of the triggering factors; however, this only occurs in about 20% of the preterm rabbit pups [115,116], limiting the utility of the spontaneous model. 

In order to increase the number of rabbits with GM-IVH, intraperitoneal administration of glycerol has been regularly used in preterm pups [107,122,150]. Glycerol can cause intravascular dehydration, increase serum osmolality and consequent decrease of intracranial pressure leading to the rupture of fragile vessels [68,107]. With this approach, premature rabbits are usually born by cesarean section at 28–29E. Glycerol is administered intraperitoneally a few hours later [63,107,108,117,119] and GM-IVH is detected in up to 95% of the rabbits [107]. Postmortem studies have shown reduced white matter myelinization and magnetic resonance imaging of the brains reveals a reduction in fractional anisotropy and white matter volume, accompanied by ventriculomegaly as well as stretching and thinning of the cortex [117]. These observations have been later confirmed and reductions in myelin basic protein have been detected in the corpus callosum and corona radiata from rabbits with GM-IVH [118]. Also, cerebellar alterations are observed after systemic glycerol administration, affecting cerebellar volume, foliation, and proliferation in a dose-dependent manner. Other studies have also reported increased neuronal apoptosis after glycerol administration to preterm rabbits [120], accompanied by cellular infiltration and neuronal degeneration in the periventricular area [119]. In line with these observations, apoptosis and axonal damage, revealed by beta-amyloid precursor protein and neurofilament immunolabeling, are also observed around the ventricles after glycerol administration [107]. On the other hand, previous studies have reported a large amount of extracellular hemoglobin deposited in the periventricular white matter in his GM-IVH model, supporting the contribution of hemoglobin to brain damage, as observed in patients [151] (see Section 2.2 and Section 2.3).

Brain pathological features are closely associated with behavioral alterations after glycerol-induced GM-IVH and previous studies have reported that 25% of pups present motor impairment with hypertonia [117] (Table 1). Pups are reported to look normal, and motor and sensory alterations are limited at 24 and 72 h. However, poor activity is observed in GM-IVH rabbits, and about 13% of the pups that develop severe hemorrhages have seizures [107]. Also, later behavioral assessment reveals that at P14, glycerol-treated rabbits present weakness in extremities, abnormal gait and limited walking speed [117]. Hind legs, righting reflex or locomotion on 30° inclination are impaired [122], whereas visual and sensory activities seem to be preserved [117] (Table 1).

Some authors have questioned the model due to the direct toxicity of glycerol in different organs, as well as the limited effect of glycerol to reproduce GM-IVH outcomes [150]. Nevertheless, the major shortcoming of this model is the diffuse character of the bleeding, that can affect different brain compartments and provoke subarachnoid, subdural, deep white substance or cortical basal ganglia hemorrhages sometimes [152]. 

#### 3.2.2. Blood and Blood Derivates-Induced GM-IVH in Rodents 

Intraventricular administration of blood or hemoglobin has been commonly used to induce GM-IVH to rats and mice [89,109,110,111,123,129]. Lesions are caused at early stages of life, ranging from P1-P7 approximately [124,127,153], when rodents brains development resembles the PTNB brain. Whereas most of the studies use maternal blood [109,128,130] or blood from other animals [88,127], some experiments have used autologous blood to induce GM-IVH both to rats [153] and mice [126,154]. Autologous blood administration has some advantages, including the lack of confounding factors, such as exogenous proteins, the possibility to study natural coagulation and inflammation pathways after spontaneous hemorrhages [155,156], or the avoidance of non-physiological substances with potentially misleading consequences [126]. However, the technical approach is especially complicated in newborn pups. In addition, whereas this approach rapidly induces an hematoma, it does not seem to induce the actual rupture of the brain blood vessels [156]. Nevertheless, the outcomes of different experimental approaches are quite similar, independently of the method and the animal model.

As commonly observed in patients, blood-induced lesions regularly result in GM-ventricle damage that includes posthemorrhagic ventricular dilatation [88,124,125,126,127], that may affect over 65% of the pups [89]. Other studies have reported posthemorrhagic ventricular dilatation in up to 90% of the animals, depending on the severity of the lesions [88], parenchyma disruption around the GM [126], loss of periventricular white matter [89] and ependymal alterations [89], including ependymal nodules with iron-laden macrophages and subependymal rosettes [88]. In line with these observations, ventricle enlargement is also observed after hemoglobin injection to neonate rats [123]. Interestingly, some studies have reported hemosiderin accumulation in the periventricular areas in P21 rats, after intraventricular blood lesions [89] as well as hemosiderin staining in the ventricular system [88], supporting the implication of blood derivates in the brain damage observed after GM-IVH. Other approaches have reported that only lysed red blood cells or iron lesions result in ventricular enlargement, whereas packed red blood cells do not [157]. Demyelination is also commonly observed, and previous studies have shown that bilateral lesions induced by maternal blood to P4 rats may result in corpus callosum thinning and cell death, accompanied of reduced myelinization in the long term (P32) [124]. Reduced myelin basic protein has also been corroborated in a similar model and experimental timing, accompanied by cell death in the periventricular area [109,111]. Similar outcomes have been observed in P5 mice injected with autologous blood, showing poor myelinization and white matter compromise at P23 [125], or in neonate rats treated with hemoglobin [123]. Apart from direct ventricular damage and demyelination, blood lesions may affect cell proliferation in the lesion area, the subventricular zone (SVZ). Controversial results have been observed at this level and whereas it has been observed that cell proliferation in the GM was bilaterally suppressed from 8 h to 7 days after autologous infusion of blood to P1 mice [141], Dawes et al. have more recently described that autologous blood administration to P0 mice induces a transient increase of proliferative cells in the ventricle wall at P4. Nevertheless, this burst of glial progenitor cells in the SVZ neurogenic niche does not seem to integrate within the cortex [126]. Neuronal loss in the hippocampus, after severe GM-IVH damage, is also accompanied by reduced cell proliferation and neurogenesis (assessed by BrdU and nesting immunostaining, respectively) in the dentate gyrus of the hippocampus [128]. In line with these observations hemoglobin-induced lesions also lead to hippocampal neuronal loss and reduced hippocampal volume [129]. Other studies have reported a reduction in the corpus callosum thickness after blood-induced GM-IVH to rats [88,121]. Further assessment also reveals that lesion-induced damage is observed all over the brain and cortical development is affected [126]. Blood breakdown products, such as hemosiderin, can also be detected in the cortex, as an indication of cortical infarcts, far from the injection site [88]. BBB leakage [124] and flattening of the choroid plexus [88] are also observed after blood-induced lesions. 

Histopathological alterations observed after blood-induced GM-IVH also result in behavioral deficits. Previous studies have reported anxiety-like behaviors in the open field [125] as well as learning and memory alterations in the passive avoidance test and the Y-maze [128]. However, most of the studies have been directed towards the analysis of motor activity, since over two-thirds of infants with progressive posthemorrhagic ventricle dilatation are known to develop motor deficits [89]. The negative geotaxis test is commonly used to assess motor disabilities in these models and, whereas some discrepancies are observed, an overall impairment is detected at different time points [88,109,111,127]. Other frequent motor abnormalities include difficulty to perform in the rotarod test [121,124] or the grip traction test [89] (Table 1). 

#### 3.2.3. Collagenase-Induced GM-IVH in Rodents 

Intraventricular administration of collagenase has also been largely used to reproduce GM-IVH in neonate rats and mice (≈P7) [32,112,149]. Bacterial collagenase is a protease that lyses the extracellular matrix, causing the rupture of the cerebral blood vessels [156]. Collagenase administration is accompanied by an inflammatory response that occurs at the site of injection [158], similar to that observed in the blood infusion model [154] and it may also induce an ischemic cerebral injury [156]. On the other hand, collagenase models can be used in different species and they are easy to reproduce. Also, induced damage is dose-dependent and specific lesion sites and timing can be controlled in the experiments. 

In 2010, Alles et al. [132] compared unilateral and bilateral collagenase lesions in P6 rats. Brain volume was significantly reduced after bilateral lesions in the short term (P7), and this effect was observed both after unilateral and bilateral lesions in the long term (P30). Ventricle enlargement and brain atrophy have been largely reproduced both in rats [133,134,135] and mice [32] after collagenase injection in the ventricles or the proximal ganglionic eminence, reproducing the pathology of the GM-IVH in the PTNB. Hydrocephalus is also commonly observed in this model [135,136,139,140] and BBB permeability, as well as implicated mechanisms, have been addressed in detail in this model (Table 1). Evans blue extravasation assay shows significant BBB leakage after collagenase injection to P7 rats [147,159]. Rolland et al. have observed alterations in markers of BBB integrity, such as decreased ratios of pAkt/Akt and GTP-Rac1/Total-Rac1 as well as the reduced expression of ZO1, occluding or claudin-3 [140] and other studies have shown similar outcomes [147]. A recent study suggests that characteristic hydrocephalus and ventriculomegaly are derived from a decrease of CSF transport through the glymphatic system, mediated by aquaporin 4 [135]. Other studies have also reported increased thrombin activity after collagenase lesions, supporting its role in hydrocephalus formation [160]. Short and long-term neuronal death is observed [32], and accumulation and upregulation of iron-handling proteins are also observed around the lesions [133]. However, the effects of the collagenase lesions are also detected in distant regions and cortical thinning [32,131,136,137,138] as well as cortical tau hyperphosphorylation have been observed [32]. General affection of the brain is supported by the widespread presence of microhemorrhages in the cortex, hippocampus and striatum from P7-lesioned mice the long term (P70) [32] and white matter lesions and basal ganglia loss are also detected after collagenase lesions [131]. In addition, neurogenesis is severely compromised in the SVZ. Likewise, reduced doublecourtin immunostaining is observed in the cortex and the hippocampus [32]. The extracellular matrix is affected by collagenase-induced GM-IVH and increased extracellular matrix protein proliferation is detected in the long term [131]. Interestingly, collagenase-induced lesions also result in altered feasible peripheral markers of the GM-IVH, including ubiquitin carboxy-terminal hydrolase L1 or gelsolin, as observed in patients, supporting the clinical relevance of this model [32].

Extensive collagenase brain damage also translates in significant behavioural impairments in different tasks. General developmental delay as well as cognitive and motor disabilities are regularly observed [134]. Eye opening latency is delayed in rats with GM-IVH and the frequency and duration of grooming and rearing are reduced [146]. Motor activity is severely compromised when animals are assessed in the righting reflex, negative geotaxis or rotarod tests. These limitations have been observed both in the short and the long term [112,135,147,148] and similar limitations have been observed when sensory motor function is assessed in rats with the composite neuroscore or foot fault tests [72,140]. As it could be expected, when unilateral and bilateral collagenase lesions are compared, ambulation, surface righting and negative geotaxis outcomes are more severely affected in bilaterally infused rats [132]. Alterations in the open field test have also been detected in rats after collagenase lesions [72,146]. In line with these observations, long-term memory deficits have been largely documented in rats analysed in the Morris water maze [72,135,140,149]. Similar outcomes have been reported in mice, when cognition is assessed in the Morris water maze or the new object discrimination test [32], showing an overall behavioural compromise, as observed in patients (Table 1). 

### 3.3. Neuroinflammation and Microglia in Animal Models of GM-IVH

GM-IVH triggers an important neuroinflammatory response, which results in a secondary brain injury that may ultimately underlie the long-term neurological deficits [146]. Therefore inflammation and anti-inflammatory therapies have been widely assessed in many previous basic science studies. Since transgenic models have limited life expectancy, studies on related neuroinflammation are scarce. Nevertheless, the 2 most up-regulated transcription factor genes in mice overexpressing VEGF in the GM are ETS1 and hypoxia-inducible factor 2α, implicated in vascular inflammation. Moreover, ToppGene analysis shows that inflammatory responses are up-regulated in bitransgenic embryos [93]. 

The inflammatory process in lesion-induced animals has been studied in depth. Whereas some differences might be observed when the etiology and experimental approaches are compared, as described in adult models [154], the neuroinflammatory response seems to be reproducible in GM-IVH models. Interestingly, while an overall exacerbated inflammatory response is observed after local lesions [32,130,142] or systemic glycerol administration [63,108] it is noteworthy that pharmacological depletion of microglia before neonatal stroke to P7 rats, significantly increases lesions and the incidence of intraparenchymal hemorrhage. Activated microglia become an important source of TGFβ1 in the injured neonatal brain, that contributes to neurovascular protection, serves as a survival factor for cerebral capillaries and contributes to the stability of the BBB [58]. These observations support a dual role for microglia and suggest that neonatal microglia may have unique functions. 

Previous studies have shown that both blood- [130] and collagenase-induced [32] lesions provoke reactive gliosis both in the short and the long term [141]. This resembles observed alterations in patients, since white matter is severely affected by GM-IVH and it is also extremely sensitive to inflammation and oxidative stress [123]. Increased neutrophil infiltration [107], increased microglia and astrocyte burdens [109,111,112,125,130,142], gliosis and glial scarring are commonly observed in the periventricular area [89,125,143]. However, increased microglia burden is also observed in brain regions distant from the lesion site, such as the cortex [32,107] or the hippocampus [144], supporting an overall inflammatory response that affects the whole brain [123]. It has also been reported that those animals that develop hydrocephalus present a much more severe gliosis that those without hydrocephalus [89]. Besides, it has been suggested that microglia may play an important role in initiating the immune response, while astrocytes may be involved in the later propagation of the inflammatory process [130]. Whereas microglia phenotype classification remains controversial, other preclinical studies show that GM-IVH interfere with M1/M2 balance and the expression of pro-inflammatory cytokines [145]. Following this idea, lesion-induced GM-IVH results in increased levels of pro-inflammatory cytokines, including IL-1β, IL-6 or TNF-α [109,145]. Other studies have also reported the implication of IL-17A and IL-17AR in GM-IVH pathology due to their role in inflammation and BBB breakdown after stroke. IL-17A and IL-17AR levels are increased, contributing to endogenous reduction of silent information regulator 1 expression and increased cell proliferation markers after collagenase-induced GM-IVH in rats [138]. Similar outcomes have been observed after systemic glycerol administration to neonate rabbits, that show increased reactive microglia and increased levels of pro-inflammatory and chemotactic effector molecules, including IL-1β, IL-6 or TNF-α, IL-8 or MCP [30,63,108]. Likewise, an upregulation of mRNA receptor genes for TLR-4, IL1R1, FAS or the transcription factor NF-Kβ is detected [120]. Whereas some studies have reported that inflammatory cytokines might be upregulated mostly in the first few days after the lesions [121], other studies have shown long-term effects of the inflammatory process in different models [32,144].

GM-IVH models have been used to further study feasible pathways responsible of the inflammatory alterations. It has been shown that GM-IVH inflammation might be mediated through AMPA receptors [63]. Also, the cannabinoid 2 receptor might mediate inflammation, since its expression is upregulated 24 h after collagenase-induced GM-IVH [161]. Neuroinflammation might be mediated by CAMKK2/AMPK/Nrf2 signaling [145] and GM-IVH-mediated endothelial nitric oxide synthase inhibition might also contribute to GM-IVH inflammation [146]. The inflammatory process has also been shown to be mediated by IFNAR/JAK1-STAT1/TRAF3/NF-κB signaling pathway in neonate rats after collagenase or blood-induced lesions [112,130] role of the L-17RA/(C/EBPβ)/SIRT1 pathway has been described as a feasible mediator of the inflammatory response observed after GM-IVH injury [118] and other studies have reported the implication of the OX-2 membrane glycoprotein via the tyrosine kinase 1 pathway [147]. On the other hand, hialuronic acid, as part of the extracellular matrix, accumulates after GM-IVH and regulates inflammation through CD44 and TLR2/4 receptors [118]. Blood and blood derivates directly trigger an inflammatory response and hemoglobin and metahemoglobin strongly correlate with TNF-α levels in neonate rabbits with GM-IVH [54]. Inflammation is followed by oxidative stress [123], and oligodendrocite damage and myelinization alterations are considered a direct consequences of GM-IVH induced inflammation [30,63]. Besides, reactive astrogliosis impairs neurogenesis and may mediate observed neuron reduction in the hippocampus after collagenase lesions [128] (Table 1). 

Inflammation is one of the most relevant pathological features associated to GM-IVH and it has been suggested that anti-inflammatory therapy for GM-IVH should start early to protect a very sensitive developing white matter [123]. Therefore, animal models have been widely used to assess therapeutic approaches for a population in great need to new alternatives. In this sense, studies focusing on the effect of anti-inflammatory drugs have also contributed to the discovery/characterization of the mechanisms that underlie the above described molecular pathways [136,144,145].

## Figures and Tables

**Table 1:** Complications associated with GM-IVH induced by glycerol, blood and blood derivates and collagenase lesions.

GM-IVH Lesion	Brain Atrophy and Myelinization	Inflammation	Motor Activity and Cognitive Impairments
Glycerol	↓ White matter, myelinization and myelin basic protein [117,118]. Ventriculomegaly [117]. Cortical thinning [117]. ↓ Cerebellar volume [118]. Neurodegeneration [119].	↑ Microglia burden (107). ↑ Pro-inflammatory cytokines [30,54,63,108,120,121].	Hypertonia [117]. ↓ Walking speed [117]. Weakness [117]. Abnormal gait [117]. Impaired locomotion on 30° inclination [122].
Blood and blood derivates	↓ White matter, myelinization and myelin basic protein [89,109,111,123,124,125]. Ventricle dilatation [7,88,89,123,124,125,126,127]. Cell death and neuronal loss [109,111,126,128,129].	↑ Reactive gliosis, microglia and astrocyte burdens [89,109,111,123,125,130]. ↑ Proinflammatory cytokines [109]. ↑ Neutrophil infiltration [107].	Altered negative geotaxis test [88,109,111,127]. Altered rotarod test [121,124] Altered grip traction test [89]. Altered open field test [125]. Memory alterations in the passive avoidance test and the Y-maze [128].
Collagenase	White matter lesions [131]. ↓ Brain volume [32,132,133,134,135]. Ventricle enlargement [32,133,134,135]. Cortical thinning [32,131,136,137,138]. Neuronal death [32]. Hydrocephalus [135,136,139,140].	↑ Reactive gliosis, microglia and astrocyte burdens [32,112,141,142,143,144]. ↑ Proinflammatory cytokines [145].	Delayed eye opening latency [146]. Altered righting reflex, negative geotaxis or rotarod tests [112,132,135,147,148]. Altered composite neuroscore and foot fault tests [72,140]. Altered open field test [72,146]. Altered Morris water maze test [32,72,135,140,149]. Altered new object discrimination test [32].

↑ increase. ↓ decrease.

## Abbreviations

BBB	Bloodbrain barrier
CNS	central nervous system
CSF	cerebrospinal fluid
GM	germinal matrix
GM-IVH	germinal matrix-intraventricular hemorrhage
PTNB	preterm newborn
TLR	Toll-like receptor
VEGF	Vascular endothelial growth factor
